# Bioinformatics and Biosimulations as Toolbox for Peptides and Peptidomimetics Design: Where Are We?

**DOI:** 10.3389/fmolb.2020.00066

**Published:** 2020-05-05

**Authors:** Ilda D’Annessa, Francesco Saverio Di Leva, Anna La Teana, Ettore Novellino, Vittorio Limongelli, Daniele Di Marino

**Affiliations:** ^1^Istituto di Chimica del Riconoscimento Molecolare, CNR, Milan, Italy; ^2^Department of Pharmacy, University of Naples Federico II, Naples, Italy; ^3^Department of Life and Environmental Sciences, New York-Marche Structural Biology Center (NY-MaSBiC), Polytechnic University of Marche, Ancona, Italy; ^4^Faculty of Biomedical Sciences, Institute of Computational Science, Università della Svizzera Italiana (USI), Lugano, Switzerland

**Keywords:** peptides design, peptidomimetics, binding free-energy, protein–protein interaction, bioinformatics tools

## Abstract

Peptides and peptidomimetics are strongly re-emerging as amenable candidates in the development of therapeutic strategies against a plethora of pathologies. In particular, these molecules are extremely suitable to treat diseases in which a major role is played by protein–protein interactions (PPIs). Unlike small organic compounds, peptides display both a high degree of specificity avoiding secondary off-targets effects and a relatively low degree of toxicity. Further advantages are provided by the possibility to easily conjugate peptides to functionalized nanoparticles, so improving their delivery and cellular uptake. In many cases, such molecules need to assume a specific three-dimensional conformation that resembles the bioactive one of the endogenous ligand. To this end, chemical modifications are introduced in the polypeptide chain to constrain it in a well-defined conformation, and to improve the drug-like properties. In this context, a successful strategy for peptide/peptidomimetics design and optimization is to combine different computational approaches ranging from structural bioinformatics to atomistic simulations. Here, we review the computational tools for peptide design, highlighting their main features and differences, and discuss selected protocols, among the large number of methods available, used to assess and improve the stability of the functional folding of the peptides. Finally, we introduce the simulation techniques employed to predict the binding affinity of the designed peptides for their targets.

## Introduction

Year by year the use of theoretical approaches to study structural and dynamical features of macromolecules (Di Marino et al., 2014, 2015a; Orozco, 2014; D’Annessa et al., 2018, 2019a) is constantly growing, thanks to the continuous improvement of methodologies and algorithms, as well as of the high performance computing facilities. Theoretical methodologies are achieving an increasing importance in many fields of science and have now gained a primary role in drug design. Indeed, hundreds of examples exist in which the use of computational techniques was crucial to discover new molecules active against different diseases (Sliwoski et al., 2014; D’Annessa et al., 2019b). In the modern era, computer-aided drug design is successfully exploited not only to develop small molecules but also to guide the more challenging design of larger size compounds like peptides or peptide-like molecules (i.e., peptoids or peptidomimetics), which can retain the physicochemical features of bioactive proteins or polypeptide chains. One such feature is the conformational plasticity of peptides that allows them to interact with larger and more shallow surfaces compared to the typically cryptic binding pockets targeted by small molecules (Di Marino et al., 2015b; Vercelli et al., 2015; Di Leva et al., 2018). Therefore, peptides and peptidomimetics represent ideal candidates for targeting protein–protein interactions (PPIs). Indeed, PPIs have emerged as relevant drug targets since they are responsible for numerous cellular processes (Wanner et al., 2011; Otvos and Wade, 2014; Sun, 2016). Nonetheless, most PPIs were until recently considered “undruggable” by small compounds due to the involvement of large binding surfaces where the recognition is ruled by both the physicochemical properties and the shape of the interacting proteins (Bakail and Ochsenbein, 2016). Similar to protein-(small)ligand interactions PPIs are stabilized by non-covalent interactions, but with hydrophobic contacts, usually responsible for recognition and packaging, playing a primary role in stabilizing the complex (Tan et al., 2016). Moreover, upon the formation of macromolecular complexes new pockets can be formed at the interface between two or more proteins, and in some cases their targeting, aimed at stabilizing, instead of disrupting, the complex, can represent a clever therapeutic strategy to treat different diseases. Also in this case, however, small compounds are often not suitable for this purpose, while peptide-like molecules are particularly favored (Henninot et al., 2018; Lee et al., 2019). Furthermore, isolated peptides can compensate for the absence of the whole protein, as in the case of hormones, or can counteract the immune system in autoimmune diseases (Lau and Dunn, 2018). Moreover, peptides have peculiar characteristics that represent advantages in the field of drug development with respect to small molecules. For instance, they show a very low or null toxicity compared to synthetic compounds, being typically degraded in non-toxic metabolites, and are highly selective against a specific target, thus making their use particularly favored (Smith et al., 2019). Finally, many peptides can be easily conjugated either to nanoparticles for targeted delivery (Valcourt et al., 2018; Kalmouni et al., 2019) or to organic molecules working as biomarkers for diagnostic purposes (Wang and Hu, 2019).

In this perspective, much effort was dedicated in the last decades to develop theoretical approaches for the design of therapeutic peptides/peptidomimetics, leading to a new branch of drug development, known as computational peptidology (Zhou et al., 2013). These strategies gave birth to a leading industry producing nearly 20 new peptide-based clinical trials annually. At the time this review was written, more than 400 peptide drugs were under clinical development and over 60 already approved for clinical use in the United States, Europe and Japan (Lee et al., 2019). Several designed peptides have shown great potential for the treatment of different types of cancers (Marqus et al., 2017; Zanella et al., 2019). Although these peptides have an extraordinary effectiveness in cancer cell cultures, they still do not provide encouraging results *in vivo* (Marqus et al., 2017). This because peptides may suffer from poor metabolic stability and membrane permeability, rapid proteolysis and unstable secondary structure (Zhang et al., 2018). With the aim to overcome such limitations, many strategies have been developed that rely on the application of chemical modifications such as cyclization, *N*-methylation, stapling or the introduction of amide bond bioisosters and non-natural amino acids. In addition, peptidomimetics can represent a valid alternative to target PPIs. Peptidomimetics are indeed organic molecules featuring physicochemical and structural properties resembling those of classical oligopeptides (Vagner et al., 2008; Zhang et al., 2018) but generally endowed with improved pharmacokinetic profiles.

The possibility to rationally design peptide-based molecules exploiting the structural characteristics of PPIs represents an enormous advantage to achieve the desired effect on the pathological process. The growing number of 3D structures available from X-ray diffraction and NMR has augmented our knowledge on protein–protein recognition and binding process, providing unprecedented insight into the proteins’ structures in the apo form states and in protein–protein and protein–peptide complexes. This information is instrumental in the peptide design process. In this perspective, combining bioinformatics approaches with molecular simulations is a valuable strategy to obtain good drug-candidate peptides. Moreover, the increased accuracy in the calculation of binding free energy allows further characterizing the energetics of the molecular binding interaction, increasing the success rate of the design process (Torrie and Valleau, 1977; Di Marino et al., 2014, 2015b; Kilburg and Gallicchio, 2016). However, the field of peptides design and PPIs prediction/refinement is really extensive and the number of approaches developed for these purposes is constantly growing. Here we provide a concise report of selected computational protocols for peptides/peptidomimetic design, paying particular attention to the most widely employed bioinformatics tools and facilities and docking algorithms available to this end. We also introduce the simulations techniques used to validate protein–peptide complexes obtained by docking procedures and to predict the binding affinity of the designed peptides for their targets.

## Peptide Design and Docking

Since PPIs emerged as druggable targets much effort was dedicated to develop algorithms and tools for peptides/peptidomimetics design. However, this is far from being a fully addressed issue and still poses many hurdles. Indeed, notwithstanding the increasing structural information available, the investigation of protein–peptide recognition is not an easy task to handle and shows several layers of complexity. For a full description of the process: (1) the three-dimensional structure of the investigated protein–protein complex should be available, in order to detect the protein region to use as a template for the design of peptides; (2) in the case the complex is not available, the protein surface that has to be recognized by the PPI disruptor should be detected, or at least predicted, with high accuracy; (3) the structure of the target protein in its apo and holo states should be known, since the binding surface might change undergoing structural rearrangement upon protein or ligand binding; (4) since peptides are highly flexible entities, their conformational flexibility, stability in solution and the ability to achieve and maintain a well-defined active structure should be considered; and (5) finally, a putative structure of the designed peptide in complex with the target protein should be generated, typically by docking, in order to provide a possible mechanism of binding. However, achieving an accurate docking of conformationally flexible peptides to a target protein is a challenging task as discussed in the following sections.

**FIGURE 1:**
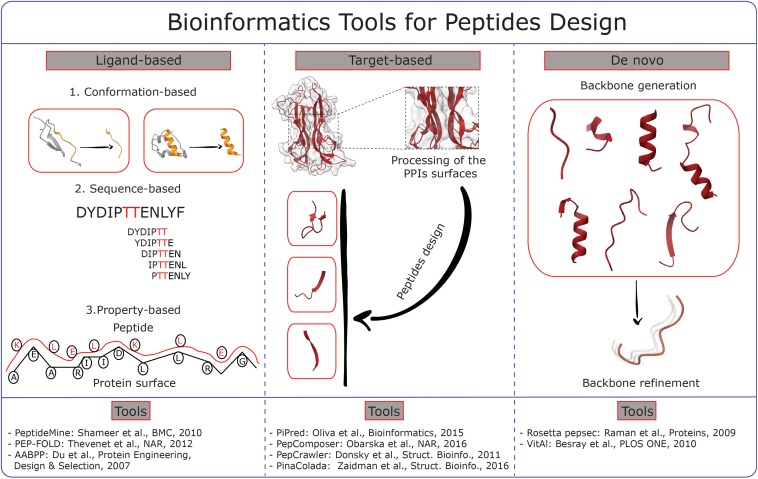
Graphical scheme summarizing different methodologies for peptide design. The core ideas of the main bioinformatics tools available are divided in three major categories: Ligand-based, Target-based and *De novo*. The references to the tools are reported at the bottom of the picture.

To date numerous bioinformatics tools for peptides design are available. These can be basically classified as ligand-based and target-based (Figure 1), even if in most of the cases the two approaches are combined. Ligand-based approaches can be further distinguished into sequence-based, conformation-based and property-based, with this last possibility still being the least explored.

Sequence-based approaches rely on the identification of conserved functional motifs, usually detected through multi-sequences alignment. These sequences are then modified to obtain a ranking of different candidates potentially able to interact with a specific target protein usually blocking an interaction with another protein partner. This is the case of the PeptideMine webserver (Shameer et al., 2010).

Substantially different are the conformation-based approaches that are aimed at building peptides structures and conformational ensembles further refined by investigation of structure-activity relationships. Example is PEP-FOLD that exploits a Hidden Markov Model to derive a structural alphabet to design stretches of “letters” that are assembled into 3D structures then refined by Monte-Carlo calculations (Thévenet et al., 2012).

Target-based strategies include knowledge-based and *de novo* design approaches. Knowledge-based methods use information from protein complexes, peptides and protein fragments (Vanhee et al., 2011). For instance, PiPred analyses protein complexes to find anchor residues and use them to find the best peptides matching the target surface from databases of fragments (Oliva and Fernandez-Fuentes, 2015). PepComposer explores a pool of protein surfaces and delivers a set of backbone scaffolds that is able to target them. A following Monte Carlo simulation refines the conformation of the newly designed peptides shown in the final peptide-protein complex (Obarska-Kosinska et al., 2016). Similarly, PepCrawler and its cognate PinaColada analyze protein complexes and derive candidate peptides that are subsequently randomly mutated in order to increase their affinity for the target. As final result, the newly designed peptides are ranked according to the predicted binding affinity (Donsky and Wolfson, 2011; Zaidman and Wolfson, 2016).

*De novo* approaches endeavor to obtain peptides without any *a priori* structural knowledge. The *pepsec* tool, included in the Rosetta suite (Raman et al., 2009), provides peptide sequences and structures that are simultaneously optimized. The process is similar to the “anchor and grow” docking algorithms in as much an anchor residue of the peptide is positioned on the protein surface and the chain is assembled starting from that point (King and Bradley, 2010). A significant advance was achieved with the implementation in Rosetta of a rotamers library that allows generating peptoid foldamers for the design of compounds with defined 3D structures thanks to the introduction of non-natural amino acids (Renfrew et al., 2014). Another example of *de novo* approaches is the VitAl algorithm, which identifies the binding site via a Coarse Grained Gaussian Network model and generates the peptides by sequentially docking pairs of residues and determining the binding energies (Besray Unal et al., 2010).

The described methodologies, especially ligand-based strategies, can be supported by stand-alone protein–peptide docking programs, in order to identify or refine the binding poses of the designed peptides. Notably, these software can be also used to predict the interaction mode of known biologically active peptides with their target, thus guiding the design of novel PPI inhibitors. Nonetheless, protein–peptide docking programs can suffer from some inaccuracies, especially in the solvation and in the conformational sampling of the ligand backbone (Zhou et al., 2013). In the last decade, however, significant progress has been made to address these issues, achieving a satisfactory quality of predictions both by knowledge-based approaches among which HADDOCK and GalaxyPepDock represent some of the most accurate software (Trellet et al., 2013; Lee et al., 2015; Van Zundert et al., 2016), and *ab initio* programs, including the newest version of the Glide SP algorithm (Glide SP-peptide) and HPEPDOCK, which exploits a hierarchical algorithm to manage peptide flexibility through an ensemble of conformations generated (Antes, 2010; Tubert-Brohman et al., 2013; Li et al., 2014; Ben-Shimon and Niv, 2015; Kurcinski et al., 2015; Schindler et al., 2015; Alam et al., 2017; Zhou et al., 2018). In HADDOCK, experimental information on the targeted PPIs is exploited to drive the docking through the inclusion of interaction restraints during the calculations. The HADDOCK procedure for flexible protein–peptide docking is a multi-step process that combines different solvent models, conformational search and selection, and induced fit algorithms in a highly efficient protocol. The GalaxyPepDock protocol consists of a combination of similarity-based docking and energy-based optimization methods. Given a target protein and a peptide, the server performs a scan of experimentally determined PPIs structures database, in order to identify a proper PPI template. Subsequently, GalaxyPepDock builds a number of protein–peptide complexes that are further refined by energy-based methods to find the best structure interface. Conversely, Glide SP-peptide, pepATTRACT or Rosetta FLexPepDock perform without any *a priori* experimental information. In particular, Glide SP-peptide relies on a grid-based docking protocol, which takes advantage of advanced sampling algorithms during the search phase. The obtained poses can be further refined by post-processing calculations with physics-based implicit solvent MM-GBSA methods, rescored and ranked by a custom scoring function. PepATTRACT combines a coarse-grained *ab initio* docking followed by an atomistic refinement protocol. In particular, a fully blind procedure is followed, where the server examines the whole protein surface to find a putative binding site and simultaneously predicts the bound peptide conformation. Finally, FlexPepDock, which is implemented in the Rosetta suite, is able to provide high-resolution protein–peptide complexes starting from a generation of coarse-grained models. These starting coarse-grained models are refined by performing Monte-Carlo Minimization restricting the peptide’s degrees of freedom and allowing the flexibility of the receptor’s binding site side chains.

## Conformational Peptide Prediction

As reported above, bioinformatics tools show a good degree of accuracy in predicting peptides conformational plasticity, mainly through internal search algorithms that iteratively build different peptide backbone conformations, each one assigned with a specific binding score. However, severe approximations still reside in the docking sampling. For instance, many docking software treat the peptide backbone as rigid during the calculations making the *a priori* knowledge of its bioactive conformation necessary. In simplest cases, when the ligand assumes a unique, or at least a prevalent conformation in water, this can be straightforwardly computed based on experimental techniques such as proton NMR experiments. This strategy can be, for instance, applied to small cyclic peptides featuring a restricted backbone conformational space. However, in many cases peptides can assume several energetically equivalent states characterized by a rugged conformational free energy landscape. In such cases, it is advisable to support the peptide design with a reliable energy estimation of the different conformations assumed by the new peptide. To this end, atomistic simulations represent a valid tool. In particular, a number of efficient conformational searching methods have been developed or specifically adapted for this purpose. These include simulated annealing (Kirkpatrick et al., 1983; Wilson and Cui, 1990), distance geometry (Donné-Op Den Kelder, 1989), random search Monte Carlo (MC) (Chang et al., 1989; Weinberg and Wolfe, 1994), eigenvector-following (Cerjan and Miller, 1981; Simons et al., 1983), basin-hopping global optimization (Wales and Doye, 1997), discrete path sampling (Wales, 2002, 2004) and molecular dynamics (MD) based algorithms. Extensive reviews are available in literature on the application of simulated annealing (Bernardi et al., 2015) and distance geometry (Mucherino et al., 2013) to study peptides conformational sampling. For this reason, here we will mainly focus on the other approaches.

Among stochastic or random search approaches is the Monte Carlo Multiple Minimum (MCMM) method, commonly known as torsional sampling (Saunders et al., 1990), in which the peptide torsional bonds are randomly rotated through iterative Monte Carlo simulations, each followed by energy minimization, in order to identify local minima in the conformational potential energy surface (PES).

An interesting example of eigenvector-following method is the low mode conformational search (LMCS) (Kolossváry and Guida, 1996), in which local minima in the PES are found through movements along the “low energy eigenvectors” that are identified through a preliminary normal mode analysis, and following energy minimization. The process is then iteratively repeated to find additional minima, eventually leading to the identification of a minimum energy path. In order to improve the performance of LMCS in global searches, a mixed MCMM/LMCS strategy has been also developed (Kolossvàry and Guida, 1999) and successfully applied to the conformational sampling of macrocyclic compounds (Parish et al., 2002).

In basin-hopping global optimization (BHGO), the potential energy landscape is transformed into a series of “basins of attraction” which are explored through a hybrid random search-geometry optimization protocol (Li and Scheraga, 1987; Wales and Doye, 1997). In detail, random structural perturbations such as backbone Cartesian moves or rotations of amino acid side chains are initially applied to the biomolecule. After each perturbation, a geometry optimization cycle is performed to find the nearest local minimum, usually through the quasi-Newton L-BFGS (Limited-memory BFGS) minimization algorithm (Liu and Nocedal, 1989). The transition is finally either accepted or rejected based on a Metropolis criterion. The method allows crossing high barriers that separate the different energy basins, thus leading to the identification of the global minimum. Also, the thermodynamic properties of the system can be computed using the data set of local minima found during the search. Many variants of the technique have been developed to specifically address problems of biological interest including peptides’ conformational sampling. For instance, the efficiency of basin hopping can be improved by including experimental restraints (Carr et al., 2015) or by combining the method with other approaches, such as parallel-tempering (Strodel et al., 2010; Joseph and Wales, 2018). Connected to BHGO, is the discrete path sampling approach. Here, a discrete path is defined as a connected sequence of minima and the intervening transition state(s) between them, which are appropriate for describing dynamical properties but can also be subjected to kinetic analysis (Wales, 2005). Discrete path sampling has been successfully used to explore the conformational energy landscape of both linear and cyclic peptides (Evans and Wales, 2004; Oakley and Johnston, 2013).

**FIGURE 2:**
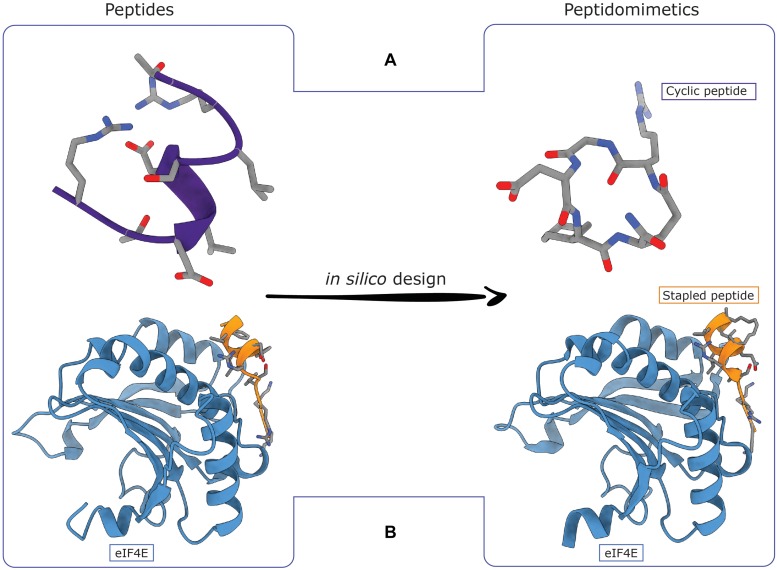
Computational strategies to transform peptides in peptidomimetics. **(A)** A metadynamics-driven design approach was successfully used to convert a helical peptide able to interact with selective the αvβ6 integrin into a cyclic pentapeptide. **(B)** A hydrocarbon stapling strategy guided by molecular dynamics (MD) simulations was enabled to successfully convert an eIF4G-derived peptide of two helix turns in a stapled peptide able to inhibit the activity of the eIF4E (PDB IDs: 4AZA and 4BEA).

Molecular dynamics (MD) based techniques are largely explored for peptides conformational sampling both as stand alone tools or in tandem with experiments. It has been indeed demonstrated that the inclusion of NMR data such as chemical shifts, interatomic distances or residual dipolar couplings (RDCs), as structural restraints in MD simulations can significantly improve the speed and efficiency of sampling algorithms. Ensemble or time-averaged MD represents a first example (Bonvin et al., 1994) followed by more recent advanced methodologies that integrate MD with experimental data. For instance, it was shown that, if geometrical restraints are applied to the system and averaged over simulation replicas, ensembles of conformations compatible with the maximum entropy principle are generated (Cavalli et al., 2013). This approach is known as replica-averaged restrained molecular dynamics and can offer a valid representation of the unknown Boltzmann distribution of a peptide conformational landscape (De Simone et al., 2011). Also, MD simulations can be coupled to Markov State Models (MSM) to predict the folding pathways and kinetics of polypeptides (Chodera and Noé, 2014; Husic and Pande, 2018). An efficient alternative strategy is to employ enhanced sampling methodologies, which allow investigating events that extend beyond the timescale limit of standard simulations. Important examples are umbrella sampling (US) (Torrie and Valleau, 1977) and metadynamics (MetaD) (Laio and Parrinello, 2002), which rely on the application of a bias on a set of user-defined reaction coordinates, specifically designed for the system under investigation, commonly referred to as collective variables (CVs). These methodologies can provide an accurate description of the free energy landscape underlying the process of interest. Particularly, MetaD (Laio and Parrinello, 2002) in its well-tempered variant (Barducci et al., 2008) was largely applied to conformational studies of both linear and cyclic peptides. For instance, Musco and coworkers employed MetaD to predict the bioactive conformation and the pharmacological behavior of cyclic penta- and hexa- peptides designed as RGD-integrin receptors modulators (Spitaleri et al., 2011; Simon et al., 2018). Remarkably, metadynamics can be combined with replica-exchange (RE) methods like parallel-tempering (PT) (Bussi et al., 2006) and bias-exchange (BE) (Piana and Laio, 2007) algorithms in which *n* exchangeable replicas of the systems are simulated at different temperatures and biasing different set of CVs, respectively. For instance, PT-MetaD was recently applied to predict the turn-helix conformation of a linear peptide reported as a selective ligand of the αvβ6 RGD-integrin, leading to new selective cyclopeptidic ligands with potential clinical applications (Figure 2A; Di Leva et al., 2018). Furthermore, the metadynamics performance can be improved through the inclusion of experimental data either in the user-defined CVs in a BE scheme (Granata et al., 2013) or as replica-averaged structural restraints. The latter approach is known as replica-averaged metadynamics (Camilloni et al., 2013) and is typically performed in the well-tempered ensemble (WTE) where the energy is used as CV (Camilloni et al., 2013). In alternative to CV-based techniques, other enhanced sampling methodologies such as accelerated MD (Hamelberg et al., 2004), replica exchange with solute-tempering (REST) (Liu et al., 2005) and reservoir-REMD (R-REMD) (Okur et al., 2007; Roitberg et al., 2007), have been successfully used for peptides’ conformational sampling. In accelerated MD the sampling is improved through the addition of a boost potential to the potential energy of the system (Hamelberg et al., 2004). This technique demonstrated to provide conformational ensembles for peptidic macrocycles well reproducing the available experimental structures (Kamenik et al., 2018). In replica exchange with solute-tempering, the contribution of solute–solvent and solvent–solvent energies are scaled in order to strengthen solvent interactions at elevated temperatures. As a result, only the solute is simulated at different temperatures as in traditional REMD, while the solvent is kept at original temperature in all replicas. The exchange probabilities exclusively depend on the contribution from solute atoms that generally show broader energy distributions compared to the solvent. Accordingly, a lower number of replicas is needed to cover the desired temperature range compared to standard REMD, thus saving computational time and resources (Liu et al., 2005). Finally, R-REMD is based on a classical PT scheme in which, the highest temperature replica is replaced by a structure reservoir that is pre-generated through standard MD simulations performed at the same temperature (Okur et al., 2007; Roitberg et al., 2007).

## Estimation of the Peptides/Peptidomimetics Binding Free-Energy

An accurate estimation of the protein–peptide binding affinity is important to guide key steps in the drug discovery pipeline such as the hit-to-lead and lead optimization processes. This is however, a challenging task to achieve with standard computational methodologies. For instance, docking algorithms can provide rapid qualitative information about the peptide binding modes but generally fail in accurately estimating receptor affinities due to the intrinsic approximations of the method. On the other hand, standard MD would require tens of microseconds of simulations to collect enough statistics to describe the full ligand binding process (Dror et al., 2011; Shan et al., 2011), which are rarely accessible with the current protocols and resources (Salmaso and Moro, 2018). The timescale limitation of classical MD can be overcome by means of free-energy methods, which can be grouped in three main categories: endpoint, alchemical perturbation and physical pathway methods.

Endpoint methods, which include linear interaction energy (LIE) (Aqvist et al., 2002), molecular mechanics Poisson–Boltzmann surface area (MM-PBSA) (Srinivasan et al., 1998), and generalized Born surface area (MM-GBSA) (Kuhn and Kollman, 2000), compute the binding free energy by taking the difference between the absolute free energy of the ligand in unbound and bound states, which are sampled separately. These methods, particularly MM-PBSA and MM-GBSA, offer a good balance between computational efficiency and accuracy, and can be successfully used to predict the binding affinities and identify or rescore the correct binding poses for protein–peptide systems (Weng et al., 2019). Interestingly, a dampened MM-PBSA scoring function was recently introduced in HADDOCK to further improve the predictiveness of the docking protocol and to estimate the protein–peptide binding affinity (Spiliotopoulos et al., 2016). Nevertheless, a large-scale application of endpoint approaches use is partly limited by some approximations to both the sampling and energy calculation which are mainly due to the use of implicit solvent models (Wang et al., 2019).

Alchemical methods are typically more rigorous and accurate, although suffering from the higher demanding computational cost. They include thermodynamic integration (TI) (Kirkwood, 1935), free-energy perturbation (FEP) (Kirkwood, 1935) and Bennett Acceptance Ratio (BAR) (Bennett, 1976; Shirts and Chodera, 2008). In these calculations, ligand and protein are gradually decoupled and the binding free energy computed from a thermodynamic path connecting the bound and unbound states. At each step of the alchemical path, the sampling can be alternatively performed using either MC or MD simulations, with the latter approach being the most widely utilized. Frequently, a translational restrained potential is applied along the path to control the turning off of the molecular interactions between the ligand and the protein binding site. This allows reducing the configurational space to sample between the end-points, thus enhancing the efficiency of the free energy calculation. Alchemical transformations which employ translational restraints are generally referred as to the “double decoupling method” (DDM), while those calculations in which no translational restraint is present are classified as “double annihilation method” (DAM) (Deng and Roux, 2009).

In physical pathway methods, which include steered molecular dynamics (SMD) (Izrailev et al., 1997) and US (Torrie and Valleau, 1977), the ligand and the receptor are physically separated along the binding pathway and finally the potential of mean force (PMF), and in turn the binding free energy, is computed. In SMD, an external force with tuneable spring constant and velocity is applied to pull the ligand out from the binding site. The PMF is then obtained from the average of the irreversible work minus the dissipative work of the process according to the Jarzynski non-equilibrium work theorem (Jarzynski, 1997a, b). Several independent SMD trajectories need to be carried out to provide a statistically significant calculation of the irreversible work, and, accordingly, an accurate estimation of the PMF. Also, the optimization of the pulling force can reduce the dissipative part of the work, which eventually leads to an increased calculations convergence. In US, an external harmonic bias potential is applied on a user-defined CV to physically drive the ligand from the bound state to the unbound state. The pathway is usually divided in *n* steps, commonly known as windows, in which standard MD calculations are performed in presence of the harmonic potential. The change in free energy between adjacent windows can be computed from the collected MD trajectories using different methods, with the most commonly used being the Weighted Histogram Analysis Method (WHAM) (Souaille and Roux, 2001).

Numerous successful applications of both alchemical and pathway methods are reported in literature. However, also these methodologies can suffer from some limitations such as: (1) a limited use to small-size ligands, for which relatively few conformations must be sampled and (2) the need of *a priori* knowledge of the ligand binding mode, for alchemical transformation methods; (3) an incomplete sampling of the ligand solvated state (Limongelli et al., 2012); (4) an insufficient sampling of the ligand bound state(s) in case of receptor’s large conformational changes; and (5) the presence of additional degrees of freedom important for the ligand binding/unbinding process which are neglected during the calculation (Limongelli et al., 2012; Limongelli, 2020). In addition, the binding free energy calculation typically converges slowly and might change in dependence of the ligand size and charge, thus hampering the application of such methods in studying peptide/peptidomimetics-protein interaction (Gumbart et al., 2013).

In the attempt to address these problems, many variants of these methodologies were developed over the last decades. In the field of alchemical transformations, for instance, REMD-based approaches were introduced to increase the accuracy and the convergence rate of calculations. Among these is a mixed FEP/REMD strategy that relies on accelerated MD simulations performed in a Hamiltonian replica exchange MD (H-REMD), in which *n* replicas of the system with a modified Hamiltonian are run in parallel and are exchanged according to specific acceptance criteria (Sugita et al., 2000). The FEP/REMD approach allows the ligand to escape from kinetically trapped conformations, which usually affect the efficiency of standard FEP/MD calculations (Jiang and Roux, 2010). A more recent example is Modeling Employing Limited Data (MELD)-accelerated MD in which experimentally derived constraints are applied in a temperature and H-REMD simulations framework (Morrone et al., 2017). Alternatively, a single decoupling method was proposed, in which a single alchemical calculation is performed in a H-REMD scheme using, however, an implicit solvent model (Kilburg and Gallicchio, 2018). In its original formalism, SDM (Single-Decoupling Binding Free Energy Method) relied on US simulations performed in Hamiltonian replica exchange and combined with the WHAM method for the calculation of the binding free energy. This approach is known as Binding Energy Distribution Analysis Method (BEDAM) and computes the binding constant through a Boltzmann-weighted integral of the probability distribution of the binding energy obtained in the canonical ensemble in which the ligand, while positioned in the binding site, is embedded in the solvent continuum and does not interact with receptor atoms (Gallicchio et al., 2010; Di Marino et al., 2015c).

As mentioned above, physical pathway methods are typically affected by an insufficient sampling of the ligand solvated state. A possible solution to this critical point was provided by the works of Roux and Henchman who introduced a cylindrical restrained potential in US simulations to reduce the sampling space in the unbound state (Woo and Roux, 2005; Doudou et al., 2009). Following this example, geometrically restricted potentials were introduced in other enhanced sampling methodologies such as MetaD. A recent example is Funnel-Metadynamics (FM) in which a funnel-shaped restrained potential is applied to the system along the simulation to reduce the phase space exploration by the ligand in the unbound state. This enhances the sampling of both the target binding site and the ligand solvated state, leading to a thorough characterization of the binding free-energy surface and an accurate calculation of the absolute protein-ligand binding free energy (Limongelli et al., 2013). So far, the method has been employed to study both ligand/protein and ligand/DNA systems (Troussicot et al., 2015; Moraca et al., 2017; Yuan et al., 2018; D’Annessa et al., 2019b), being suitable also in the investigation of peptide-protein binding processes.

## Concluding Remarks

Designing peptides able to interact with specific target proteins is only the first step toward the development of compounds that can be considered as drug candidates. Despite their great potential, as largely discussed above, some limitations to the use of peptides in clinical routines still exist, mainly due to their low stability in solution, poor permeability through cellular membranes and physiological barriers, such as the blood–brain barrier (BBB).

The introduction of modifications in the chemical structure that could stabilize a peptide in its bioactive conformation, increasing efficiency, represents the smartest strategy. This can be achieved by introducing non-natural side chains, D-amino acids, non-alpha-amino-acids, peptide bond isosteres, staples and cyclization that change peptides into peptoids or peptidomimetics (Figure 2; Vagner et al., 2008; Zhang et al., 2018). Typically, these modifications are designed by either adding chemical functional groups to a well-characterized active peptide or using small molecules as building blocks that mimic the amino acids backbone with the aim of reproducing the geometry of secondary structure elements (SSE) (i.e., α-helix and β-strand) of bioactive peptides (Vagner et al., 2008; Zhang et al., 2018). Indeed, SSEs play a key role in PPIs, and among them α-helices are the most commonly found at PPI interfaces. Peptidomimetics guarantee enhanced protection against peptidases, improved systemic delivery and cell penetration, high target specificity and poor immune response and they are already in use against different pathologies, such as cancer and diabetes (Vagner et al., 2008; Zhang et al., 2018). In this context, computational approaches such as MetaD (Figure 2A) and classical MD simulations (Figure 2B) demonstrated to be valid tools to drive the conversion of peptides in more active peptoids/peptidomimetics, targeting αvβ6 RGD-integrin in one case (Di Leva et al., 2018) and the eukaryotic translation initiation factor 4E (eIF4E) in the other (Lama et al., 2013, 2019).

As highlighted in this review, peptides and peptidomimetics can play a central role in pharmacological applications, also having a potential strong economic impact on the pharmaceutical industries. Indeed, the use of peptides/peptidomimetics for the treatment of very different pathologies, including some types of cancer, Alzheimer’s disease, metabolic diseases and microbial infections, is now becoming a standard approach (Qvit et al., 2017; Mabonga and Kappo, 2019).

Furthermore, the implementation of “hybrid” approaches that combine theoretical and experimental techniques can sensibly assist drug design, allowing, for instance, to overcome some issues related to the development of peptides, mainly due to their nature and size.

We strongly believe that the improvement of computational peptidology techniques aimed at modifying and increasing the potential of these molecules to obtain multifunctional peptides, cell penetrating peptides and peptide drug conjugates, will help strengthen the efficacy and the applicability of peptides as therapeutics.

In conclusion, peptide design is an appealing but complex process that raises many challenges and for a successful outcome a deep knowledge of the available approaches and how to combine them to overcome some major drawbacks are necessary.

## Author Contributions

This mini-review article was conceived by DD with contributions from all authors, under the supervision of DD and VL.

## Conflict of Interest

The authors declare that the research was conducted in the absence of any commercial or financial relationships that could be construed as a potential conflict of interest.
